# Endurance training in early life results in long‐term programming of heart mass in rats

**DOI:** 10.14814/phy2.12720

**Published:** 2016-02-18

**Authors:** Glenn D. Wadley, Rhianna C. Laker, Glenn K. McConell, Mary E. Wlodek

**Affiliations:** ^1^ Centre for Physical Activity and Nutrition ResearchSchool of Exercise and Nutrition SciencesDeakin UniversityGeelongVictoriaAustralia; ^2^ Department of PhysiologyThe University of MelbourneParkvilleVictoriaAustralia; ^3^ Institute of Sport, Exercise and Active LivingVictoria UniversityVictoriaVictoriaAustralia

**Keywords:** Endurance training, fetal programming, heart hypertrophy

## Abstract

Being born small for gestational age increases the risk of developing adult cardiovascular and metabolic diseases. This study aimed to examine if early‐life exercise could increase heart mass in the adult hearts from growth restricted rats. Bilateral uterine vessel ligation to induce uteroplacental insufficiency and fetal growth restriction in the offspring (Restricted) or sham surgery (Control) was performed on day 18 of gestation in WKY rats. A separate group of sham litters had litter size reduced to five pups at birth (Reduced litter), which restricted postnatal growth. Male offspring remained sedentary or underwent treadmill running from 5 to 9 weeks (early exercise) or 20 to 24 weeks of age (later exercise). Remarkably, in Control, Restricted, and Reduced litter groups, early exercise increased (*P *<* *0.05) absolute and relative (to body mass) heart mass in adulthood. This was despite the animals being sedentary for ~4 months after exercise. Later exercise also increased adult absolute and relative heart mass (*P *<* *0.05). Blood pressure was not significantly altered between groups or by early or later exercise. Phosphorylation of Akt Ser^473^ in adulthood was increased in the early exercise groups but not the later exercise groups. Microarray gene analysis and validation by real‐time PCR did not reveal any long‐term effects of early exercise on the expression of any individual genes. In summary, early exercise programs the heart for increased mass into adulthood, perhaps by an upregulation of protein synthesis based on greater phosphorylation of Akt Ser^473^.

## Introduction

Babies born small for gestational age have a higher risk of developing cardiovascular disease, hypertension, insulin resistance, and type 2 diabetes in adults (Barker et al. 1989; Eriksson et al. 2000; Storgaard et al. 2006). In humans, uteroplacental insufficiency is the major cause of fetal growth restriction in developed countries (Barker et al. 1989; Eriksson et al. 2000; Storgaard et al. 2006). Our group and others have found that restriction of growth in prenatal life induced by a model of uteroplacental insufficiency adversely impacts on later cardiovascular and metabolic health including increased rates of hypertension (Vickers et al. 2000; Wlodek et al. 2007, 2008; Black et al. 2012), glucose intolerance (Siebel et al. 2008), and myocardial insulin resistance (Tsirka et al. 2001). Our group has also found that fetal growth restriction adversely impacts the heart during postnatal development, with lower cardiomyocyte number (Black et al. 2012) and reduced cardiac expression of genes involved in mitochondrial metabolism, glucose transport, and oxidative stress (Wadley et al. 2013). In normal birth weight adult rats (~6 months old), endurance training significantly increases heart mass and enzyme activity of proteins involved with antioxidant defense and mitochondrial energy production (Hickson et al. 1979, 1983; Lennon et al. 2004). Whether endurance exercise training can increase the heart mass in rats exposed to uteroplacental insufficiency has to our knowledge yet to be examined.

Moderate intensity endurance training has been shown to lower blood pressure in hypertensive patients (Fagard 2005, 2006; Fagard and Cornelissen 2007) and rats (Tipton et al. 1987, 1991). The effect of exercise to reduce blood pressure in humans and rats is partly a consequence of physiological cardiac hypertrophy resulting in increased stroke volume and reduced heart rate. There have also been varied effects reported on cardiac output and total peripheral resistance (Tipton et al. 1991; Pluim et al. 2000; McMullen and Jennings 2007) as well as possible increases in vascular compliance (Green et al. 2011). Whether endurance training can reduce adult hypertension in rats exposed to uteroplacental insufficiency is unknown.

In humans, a large scale prospective study reported higher physical activity levels before or after puberty being associated with lower prevalence of hypertension (and diabetes) in adulthood (Fernandes and Zanesco 2010). Importantly, current adult physical activity levels were not related to these outcomes (Fernandes and Zanesco 2010). Somewhat related, we recently reported that a few weeks of endurance training early in life (from 5 to 9 weeks of age) restored a ~45% deficit in pancreatic *β*‐cell mass in adulthood in growth restricted male rats (Laker et al. 2011). Together these findings suggest that early‐life exercise training can provide beneficial health outcomes in adulthood and that tissues adversely affected by fetal growth restriction can potentially be “reprogrammed” by early‐life exercise. Therefore, the aim of this study was to examine if early‐life endurance training could increase heart mass and normalize the hypertension we have previously observed in the adult heart from growth‐restricted male rats (Wlodek et al. 2008; Black et al. 2012).

## Materials and Methods

### Ethical approval

All experimental procedures were approved by The University of Melbourne Animal Experimentation Ethics Sub‐Committee and conducted in accordance with accepted standards of humane animal care.

### Animal procedures

The animals used in this study are a subset of a cohort previously published in Laker et al. (Laker et al. 2011) and in the current study we investigated the effects of exercise training on male offspring only. Briefly, Wistar Kyoto rats (9–13 weeks of age; *n* = 75 total) were obtained for breeding purposes from the Australian Resource Centre (Murdoch, WA, Australia) and provided with a 12‐h light–dark cycle with access to standard rat chow and water ad libitum.

Since we have previously shown that reductions in litter size independently programs postnatal growth restriction and hypertension in males (O'Dowd et al. 2008; Wlodek et al. 2008), all comparisons were made between offspring of sham‐operated controls of normal litter size (Controls) and a separate group of postnatal growth restriction induced by reduced litter size, which underwent sham surgery (termed Reduced litter), and litters exposed to uteroplacental insufficiency (termed Restricted), who have both prenatal and postnatal growth restriction.

On day 18 of gestation pregnant rats underwent bilateral uterine vessel ligation surgery or sham surgery under anesthesia with an intraperitoneal injection of ilium xylazil‐20 (10 mg kg^−1^) and ketamine (50 mg kg^−1^) (Restricted group; *n* = 25) (O'Dowd et al. 2008; Wlodek et al. 2008; Laker et al. 2011). At birth (day 22), half of the sham‐operated mothers (litter size 10–14) had their litter size randomly reduced to five pups (termed Reduced litter) to match that of the Restricted litters (litter size ~5) (O'Dowd et al. 2008; Wlodek et al. 2008; Laker et al. 2011). Males from the same litter were allocated to different treatment groups and up to three males were used from each litter. At 5 weeks of age, male offspring from each experimental group (Control, Restricted, and Reduced litter, *n* = 40 in each group) were allocated to one of the following exercise treatments: remained sedentary with postmortem (PM) at 9 or 24 weeks, early exercise training (from 5 to 9 weeks of age) with PM at 9 or 24 weeks, or later exercise training (from 20 to 24 weeks of age) with PM at 24 weeks (*n* = 8 males/group) (Wlodek et al. 2008; Laker et al. 2011). Body weight at day 1 was taken as the average of all males in each litter. Individual body weight was measured on days 6 and 14 and weeks 5, 9, 12, 16, 20, and 24.

### Exercise training

Exercise training involved treadmill running 5 days per week for 4 weeks as described previously (Laker et al. 2011, 2012). Running duration progressively increased from 20 up to 60 min, with the treadmill speed set at 15 m min^−1^ for the first week and 20 m min^−1^ thereafter (Laker et al. 2011, 2012).

### Systolic blood pressure measurements by tail cuff plethysmography

Systolic blood pressure was measured by noninvasive tail cuff plethysmography at 9, 16, 20, and 24 weeks of age. Tail cuff plethysmography was used as it allows for repeated measures to be made over an extended period of time in the same rat and is relatively high throughput (Wlodek et al. 2007, 2008; Black et al. 2012). Conscious rats were individually housed in a small cage (16 × 33 × 13 cm^3^) and gently warmed under mild heat lamps. The tail cuff was fitted around the upper part of the tail with the pulse transducer (ADIntruments Pty. Ltd., Castle Hill, NSW, Australia) positioned just below, directly aligned against the caudal artery. Measurements were collected 8–10 times consecutively for each rat in order to reduce the initial effects of stress on the systolic blood pressure values. Systolic blood pressure was determined as the mean of up to the last five consistent measurements. Cuff pressure and caudal artery pulse signals were logged by the digital recording system Powerlab4 (ADInstruments) connected to the software program LabChart6 (ADInstruments).

### Preparation of rat tissue

At 9 or 24 weeks of age rats were killed with an intraperitoneal injection of ilium xylazil‐20 (30 mg kg^−1^) and ketamine (225 mg kg^−1^). The rats in the 9‐week‐old early exercise and 24‐week‐old later exercise groups were killed 72 h following the last bout of treadmill running. The whole heart was rapidly excised (Wadley et al. 2013), weighed, snap‐frozen, and crushed into a powder in liquid nitrogen and then stored at −80°C for later analysis.

For analysis of total protein content, frozen heart tissue was homogenized in ice‐cold lysis buffer (20 *μ*L buffer/mg muscle; 50 mmol L^−1^ Tris at pH 7.5 containing 1 mmol L^−1^ EDTA, 10% vol/vol glycerol, 1% vol/vol Triton X‐100, 50 mmol L^−1^ NaF, 5 mmol L^−1^ Na_4_P_2_O_7_, 1 mmol L^−1^ DTT, 1 mmol L^−1^ PMSF, and 5 *μ*L mL^−1^ protease inhibitor cocktail [P8340; Sigma, St. Louis, MO]) and then the protein concentration of the homogenate was determined using the bicinchoninic acid protein assay (Pierce, Rockford, IL) and BSA was used as the standard. For immunoblotting analysis the remaining homogenate was centrifuged at 16,000 *g* for 20 min at 4°C and the protein concentration of the supernatant was determined using the bicinchoninic acid protein assay (Pierce, Rockford, IL) and BSA was used as the standard.

Total RNA was extracted from frozen heart with Trizol and DNase on‐column digestion (Invitrogen, Melbourne, Australia) as described previously (Wadley and McConell 2010).

### Immunoblotting

Total lysates were solubilized in Laemmli sample buffer. Equal amounts of total protein (20 *μ*g) were separated by SDS‐PAGE and electrotransfer of proteins from the gel to PVDF membranes. The mouse monoclonal antibody for phospho‐Akt Ser^473^ (pSer^473^ Akt) was from Cell Signaling Technology (Hertfordshire, England). Binding was detected with IRDye^™^ 680‐conjugated anti‐mouse IgG (Molecular Probes, Eugene, OR) fluorescent antibody via infrared detection (Odyssey Imaging system, LI‐COR Biosciences, Lincoln, NE). Membranes were then reprobed with rabbit polyclonal Akt antibody (Cell Signaling). Binding was detected with IRDye^™^ 800‐conjugated anti‐rabbit IgG (Rockland, Gilbertsville, PA). As a loading control, blots were reprobed with anti‐*α*‐tubulin mouse monoclonal antibody (Sigma). A three‐point standard curve (e.g., 10 *μ*g, 20 *μ*g, and 40 *μ*g of total protein from one sample) was run on every membrane to confirm a 100% increase in protein loaded resulted in a 100% increase in signal intensity for all proteins of interest (Mollica et al. 2009).

### Microarray

Whole‐genome DNA microarray including data transformation and ANOVA analysis was completed in the whole heart by the Australian Genome Research Facility (Parkville, Victoria, Australia) using the Illumina direct hybridization assay. The quality and quantity of RNA was ascertained on the Agilent Bioanalyser 2100 using the NanoChip protocol. A total of 500 ng of RNA was labeled using the Ambion Total Prep RNA amplification kit (Ambion). A total of 1.5 *μ*g of amplified RNA was then prepared for hybridization to the Ilumina Rat Expression Beadchip by preparing a probe cocktail (cRNA @ 0.05 *μ*g *μ*L^−1^) that includes GEX‐HYB Hybridization Buffer (supplied with the beadchip). A total hybridization volume of 30 *μ*L was prepared for each sample and 30 *μ*L loaded into a single array on the Ilumina Rat Expression Beadchip. A total of six different labeled samples can be loaded into six individual arrays per beadchip. The chip was then hybridized at 58°C for 16 h in an oven with a rocking platform. After hybridization, the chip was washed, coupled with Cy3, and scanned in the Illumina iScan Reader. The scanner operating software, GenomeStudio, converts the signal on the array into a TXT file for analysis.

Raw signal intensity values were subject to variance stabilization transformation including background correction, log2 transformation, and variance stabilization using the lumiR package of R Bioconductor (www.Bioconductor.org). (Du et al. 2008; Lin et al. 2008). ANOVA analysis of normalized probe intensities values was performed in Partek^®^ Genomic Suite^™^ software, version 6.6 (Partek Inc., St. Louis, MO). ANOVA was used to calculate significance of variation in normalized expression values between sample groups, fold change of gene expressions was calculated as mean ratio. Probes with an unadjusted *P* value of 0.05 or less and an absolute fold change of <0.75 or >1.25 or more were defined as differentially expressed. Gene‐set enrichment analyses (GSEA) entailing gene ontology (GO) was performed on the list of differentially expressed Illumina probes (representing genes) using Database for Annotation, Visualization, and Integrated Discovery (DAVID) v6.7 (http://david.abcc.ncifcrf.gov) (Huang et al. 2008; da Huang et al. 2009). The microarray data were prepared according to Minimum Information About Microarray Experiment (MIAME) recommendations and deposited into the National Center for Biotechnology Information Gene Expression Omnibus database (http://www.ncbi.nlm.nih.gov/geo/query/acc.cgi?acc=GSE75781) with accession number GSE75781.

### Real‐time PCR

Real‐time PCR was conducted to validate the gene expression findings from our microarray data in Control rats and to also extend our findings to the Restricted and Reduced litter rats. Four differentially expressed genes identified in the array as having >1.25‐fold expression in both early and later exercise groups (compared to sedentary) were selected for real‐time PCR analysis in 24‐week‐old rats (Table 6). In addition, cytochrome c oxidase subunit IV and creatine kinase were also selected for real‐time PCR analysis due to them being metabolic genes found to be differentially expressed in the array with >1.25‐fold expression in the later exercise group (compared to sedentary) and expected to be increased in the adult heart following endurance training (Echegaray and Rivera 2001; Qi et al. 2011).

RNA concentration was determined by spectrophotometric analysis and first‐strand cDNA was generated from 0.5 *μ*g RNA using a commercially available kit, including treatment with RNaseH (Invitrogen, Melbourne, Australia). Real‐time PCR using SYBR^®^ Green chemistry was performed as described previously (Wadley et al. 2013) using the MX3000p thermal cycler system (Stratagene, Amsterdam, The Netherlands). Primer sequences for ras homolog family member A (Rhoa, NM_057132.3) were 5′‐GGTTTATGTGCCCACGGTGT‐3′ and 5′‐TACCGGCTCCTGCTTCATTTT‐3′; for secreted phosphoprotein 1, also known as osteopontin (Spp1, NM_012881.2) were 5′‐CAGAGGAGAAGGCGCATTACA‐3′ and 5′‐AATCCTCGCTCTCTGCATGG‐3′; for creatine kinase, muscle (Ckm, NM_012530.1) were 5′‐ACAGCAAAGACAGACACTCAGG‐3′ and 5′‐GAACTTGTTGTGGGTGTTGC‐3′; for superoxide dismutase 3, extracellular (Sod3, NM_012880.1) were 5′‐CTTGGGAGAGCTTGTCAGGT‐3′ and 5′‐CACCAGTAGCAGGTTGCAGA‐3′; for cytochrome c oxidase subunit IV isoform 1 (Cox4i1, NM_017202.1) were 5′‐GTGCTGATCTGGGAGAAGAGCTA‐3′ and 5′‐GGTTGACCTTCATGTCCAGCAT‐3 and for beta actin (Actb, NM_031144) were 5′‐GACAGGATGCAGAAGGAGATTACT‐3′ and 5′‐TGATCCACATCTGCTGGAAGGT‐3′. The mRNA of each gene was normalized to the absolute cDNA content in each sample that was determined using an OliGreen assay with an oligonucleotide standard (Invitrogen) as described previously (Wadley et al. 2013). This is a suitable method of normalization that avoids the many problems associated with “housekeeping genes” (Lundby et al. 2005; Wadley et al. 2013).

### Statistical analyses

For weights, immunoblotting, and real‐time PCR measures, two‐way ANOVA was performed to determine main effects of experimental group and exercise treatment. If this analysis revealed a significant interaction, specific differences between mean values were located with Bonferroni post hoc analysis in SPSS. If a main effect was observed, Bonferroni's multiple comparisons test were performed where appropriate. Data are presented as mean ± SE and *P *<* *0.05 was considered statistically significant.

## Results

### Body and dorsal fat weights

The body weight and dorsal fat weight represent a subset of the data previously published in Laker et al. (Laker et al. 2012). The litter size was significantly lower (*P *<* *0.05) in bilateral uterine vessel ligation (Restricted) rats compared with sham‐operated Controls (5.6 ± 0.5 vs. 8.2 ± 0.7, respectively). Restricted offspring had significantly lower birth weight compared with Controls and Reduced litters (3.63 ± 0.06 g vs. 4.31 ± 0.03 g and 4.43 ± 0.04 g, respectively). Restricted offspring remained lighter (*P *<* *0.05) than Controls throughout life (Laker et al. 2012). Reduced litter offspring accelerated their growth such that they were heavier (*P *<* *0.05) than Controls from 16 weeks of age onward (Laker et al. 2012). Body weight was not significantly altered by early or later exercise (Laker et al. 2012). At 9 weeks of age, relative dorsal fat mass was significantly lower in both Restricted and Reduced litter offspring compared with Controls and there was no effect of early exercise (Laker et al. 2012).

### Blood pressure

**Figure 1:**
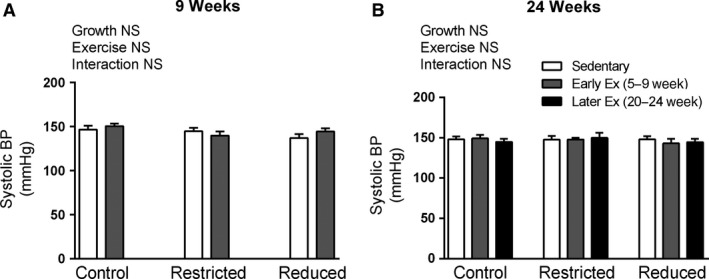
Systolic blood pressure (BP) at 9 and 24 weeks of age (A and B, respectively). Data are presented as mean ± SEM (*n* = 10–12/group). Early exercise (Early Ex) and later exercise (Later Ex).

Blood pressure was not significantly different in Restricted or Reduced litter rats compared with Controls at any age, nor was blood pressure significantly altered by early or later exercise (Fig. 1 and data not shown).

### Heart weight

**Figure 2:**
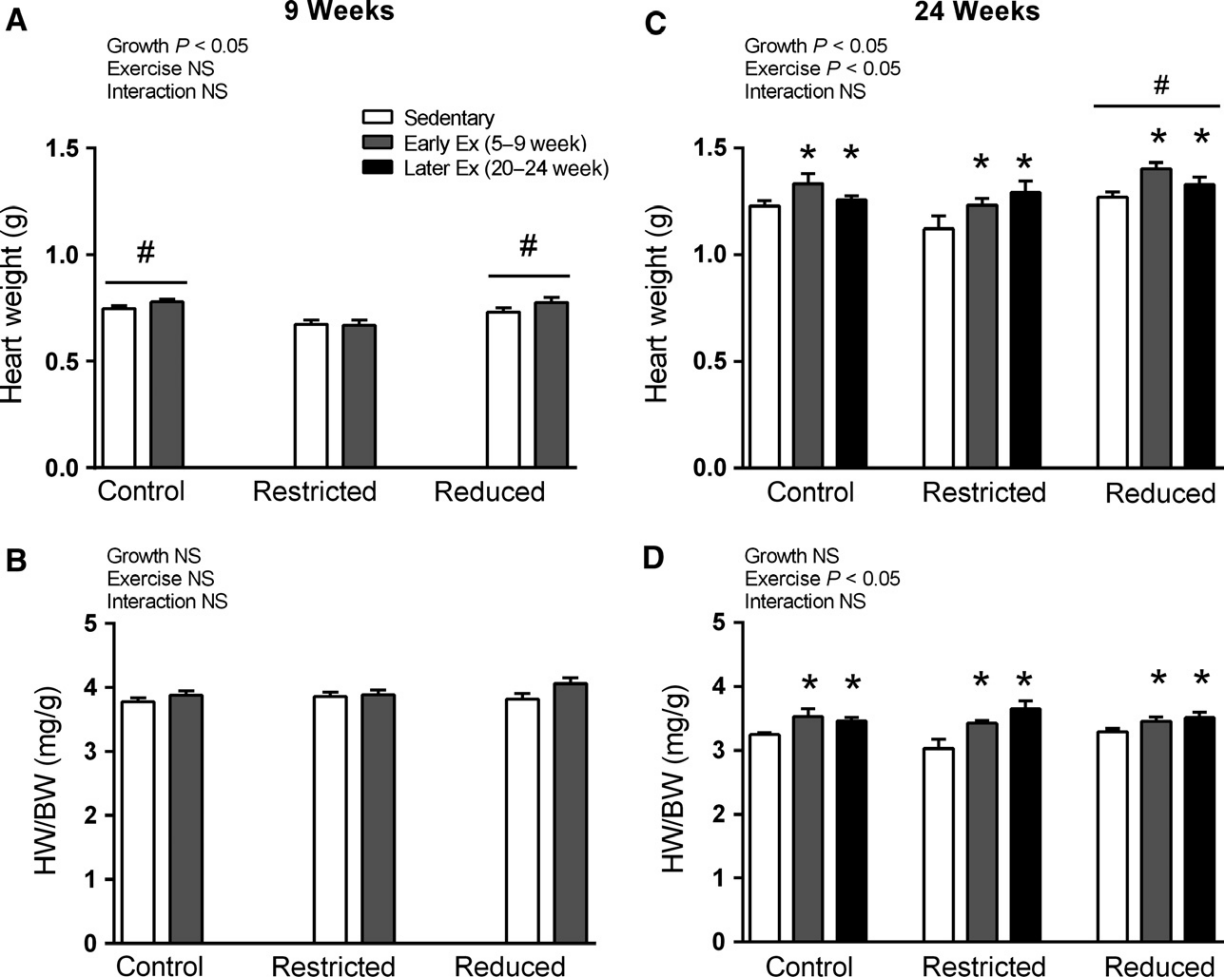
Absolute heart weight at 9 and 24 weeks of age (A and B, respectively). Relative heart weight normalized to body weight at 9 and 24 weeks of age (C and D, respectively). Data are presented as mean ± SEM (*n* = 10–12/group). Early exercise (Early Ex) and later exercise (Later Ex). *P <* *0.05 versus Sedentary*,* ^#^ *P *<* *0.05 versus Restricted (Bonferroni comparisons of main effects).

At 9 weeks of age, absolute heart weight was significantly lower in Restricted rats (*P *<* *0.05, Fig. 2A, main effect for growth), although this was due to a reduced body weight, since relative heart weight was not significantly different between Control, Restricted, and Reduced litter groups (Fig. 2B). Furthermore, early exercise did not alter either absolute or relative heart weight at 9 weeks of age (Fig. 2A and B, respectively).

At 24 weeks of age, both early and later exercise significantly increased absolute and relative heart weight (*P* < 0.05, main effect for exercise, Fig. 2C and D, respectively). The Reduced litter rats had significantly higher absolute heart weights (*P *<* *0.05, Fig. 2C, main effect for growth), although when normalized to body weight there were no significant differences between Control, Restricted, and Reduced litter groups (Fig. 2D).

### Heart protein and RNA content

At 24 weeks of age, the percentage of total protein in the heart was significantly lower in Restricted and Reduced litter rats compared to Controls (*P* < 0.05, main effect of growth, Table 1). Furthermore, neither early nor later exercise significantly altered the percentage of total protein in the hearts from Control, Restricted, and Reduced litter groups (Table 1). The RNA content in the heart was not significantly different between Control, Restricted, and Reduced litter groups, nor did early or later exercise alter RNA content (Table 1).

**Table 1:** Protein and RNA content in whole heart from 24‐week‐old rats

Group	Exercise treatment	Heart protein (% wet wt)	Heart RNA (*μ*g g^−1^ wet wt)
Control	Sedentary	16.7 ± 0.5	270 ± 25
	Early exercise	17.0 ± 0.7	281 ± 16
	Later exercise	18.5 ± 0.6	245 ± 15
Restricted	Sedentary	16.1 ± 0.5^a^	252 ± 19
	Early exercise	15.9 ± 0.5^a^	259 ± 31
	Later exercise	15.7 ± 0.5^a^	291 ± 30
Reduced litter	Sedentary	16.4 ± 1.0^a^	241 ± 28
	Early exercise	15.3 ± 0.6^a^	284 ± 36
	Later exercise	15.0 ± 0.9^a^	228 ± 21

Data presented as mean ± SE (*n* = 7–10 per group).

a *P* < 0.05 versus Control (Bonferroni comparisons of main effects).

### Akt phosphorylation and protein abundance in the heart

**Figure 3:**
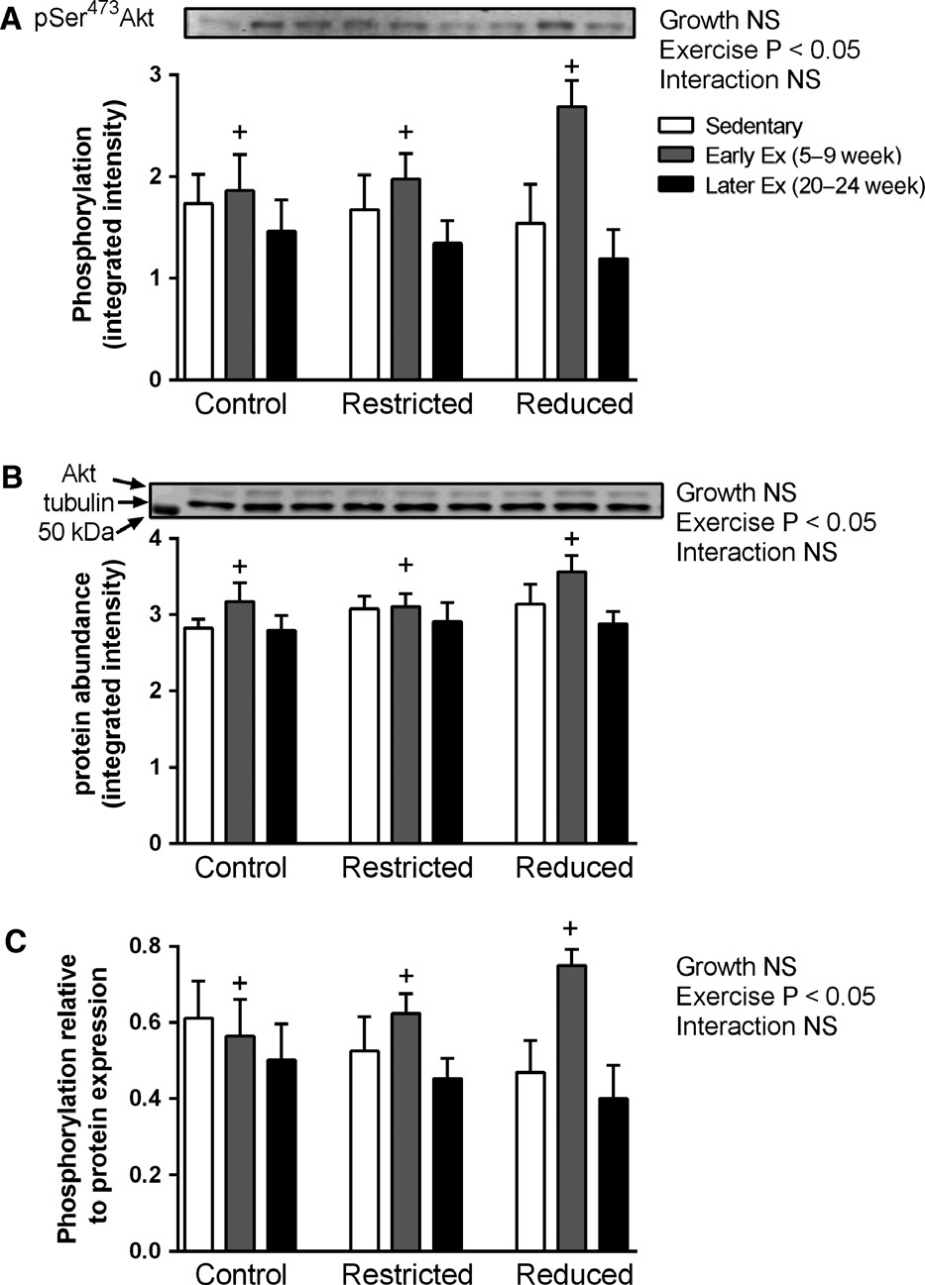
Whole heart (A) phosphorylation of Akt Ser^473^ (pSer^473^ Akt), (B) Akt (top band) and tubulin (lower band) protein abundance, and (C) pSer^473^ Akt normalized to Akt, in adult 24‐week‐old rats. Western blots are representative from one rat from each treatment group that was run on the same gel. Values are mean ± SEM;* n *=* *8 for all groups. Early exercise (Early Ex) and later exercise (Later Ex). ^+^ *P *<* *0.05 versus Later Ex (Bonferroni comparisons of main effects).

At 24 weeks of age, both the phosphorylation and protein abundance of Akt were significantly higher in the early exercise compared to the later exercise groups (*P *<* *0.05, main effect for exercise, Fig. 3A and B, respectively). Furthermore, even when normalized to Akt protein abundance, the phosphorylation of Akt Ser^473^ was significantly higher in the early exercise groups compared to the later exercise groups (*P *<* *0.05, main effect for exercise, Fig. 3C). Tubulin protein abundance was not significantly different in any group for any treatment (data not shown).

### Microarray for control rats

To investigate the molecular pathways that early exercise might be regulating the increased heart mass in adulthood we performed microarray analysis. Due to the large number of groups and because the heart mass response to training was similar in the three groups (Control, Reduced litter, and Restricted) we chose to perform microarray in the Control (sedentary, early, and later exercise) animals only. At 9 weeks of age, 21 genes were identified as being differentially expressed in sedentary versus early exercise rats, with 19 having increased expression and 2 having reduced expression following early exercise (Table 2). At 9 weeks of age, there were no gene ontology (GO) categories found to be overrepresented.

**Table 2:** List of DNA microarray differentially expressed genes in 9‐week‐old hearts from Control rats in the sedentary versus early exercise rats; >1.25 fold change. A fold change >1 indicates higher expression in sedentary versus early exercise rats. *N* = 7 rats per group

Illumina #	Gene symbol	Fold change Sedentary versus Early Ex	*P* value
ILMN_1360881	MGC112899	1.45	0.0023
ILMN_1350984	Mtch1	1.43	0.0002
ILMN_1360804	LOC364105	1.36	0.0023
ILMN_1364330	Rad23a	1.36	0.0219
ILMN_1355133	RGD1565319_predicted	1.36	0.0008
ILMN_1352142	C1qb	1.32	0.0118
ILMN_1371456	Cfd	1.31	0.0057
ILMN_1376746	Gng10	1.31	0.0008
ILMN_1350882	Eef1b2_predicted	1.30	0.0001
ILMN_1374391	Rhoc_predicted	1.29	0.0004
ILMN_1373883	S100a11	1.29	0.0007
ILMN_1374995	Srp19_predicted	1.28	0.0053
ILMN_1352889	Dynlt1	1.28	0.0001
ILMN_1363374	RGD1566189_predicted	1.28	0.0285
ILMN_1367581	RGD1560687_predicted	1.27	0.0379
ILMN_1366645	RGD1562315_predicted	1.27	0.0042
ILMN_1372866	Slc25a20	1.26	0.0014
ILMN_1373042	Sharpin	1.26	0.0015
ILMN_1374010	RGD1561310_predicted	1.26	0.0086
ILMN_1350868	Hcn2	0.75	0.0204
ILMN_1363201	RGD1563867_predicted	0.74	0.0217
ILMN_1361942	LOC689560	0.74	0.0307

At 24 weeks of age, 43 genes were identified as being differentially expressed in early exercise versus sedentary rats, with 35 having increased expression and 8 having reduced expression following early exercise (Table 3). GO annotations revealed a small number of the genes were clustered significantly (3–6 genes per GO term) to biological processes that included translational elongation, response to oxygen levels, cell migration, and negative regulation of apoptosis and positive regulation of multicellular organismal process (Table 4).

**Table 3:** List of DNA microarray differentially expressed genes in 24‐week‐old hearts from Control rats in the sedentary versus early exercise rats; <0.75 or >1.25 fold change. A fold change >1 indicates higher expression in exercise rats. *N* = 7 rats per group

Illumina #	Gene symbol	Fold change Sedentary versus Early Ex	*P* value
ILMN_1352642	Spp1	1.91	0.0126
ILMN_1349268	Rhoa	1.50	0.0003
ILMN_1355682	Cd63	1.44	0.0142
ILMN_1359425	RGD1305975_predicted	1.40	0.0018
ILMN_1351139	LOC498279	1.34	0.0001
ILMN_2038798	Actb	1.34	0.0255
ILMN_1363818	Ssr2_predicted	1.33	0.0050
ILMN_1350091	G0s2	1.32	0.0434
ILMN_1363374	RGD1566189_predicted	1.32	0.0109
ILMN_1376694	Crem	1.32	0.0001
ILMN_1367581	RGD1560687_predicted	1.32	0.0140
ILMN_1360900	Wbp5_predicted	1.32	0.0001
ILMN_1366981	Ftl1	1.32	0.0124
ILMN_1351387	RGD1565798_predicted	1.30	0.0001
ILMN_1369599	Pf4	1.30	0.0019
ILMN_1376746	Gng10	1.30	0.0009
ILMN_1365192	S100a4	1.30	0.0064
ILMN_1372199	LOC501744	1.29	0.0009
ILMN_1364180	RGD1564883_predicted	1.29	0.0015
ILMN_1651012	LOC692000	1.29	0.0028
ILMN_1373883	S100a11	1.29	0.0006
ILMN_1359331	LOC687248	1.28	0.0001
ILMN_1359173	Pigy	1.28	0.0000
ILMN_1367281	Rpl41	1.27	0.0062
ILMN_1355039	Actb	1.27	0.0489
ILMN_1650399	Tagln2	1.27	0.0084
ILMN_1359200	Timp1	1.26	0.0059
ILMN_1366973	LOC364139	1.26	0.0031
ILMN_1365031	Txndc15	1.26	0.0272
ILMN_1361581	Sod3	1.26	0.0020
ILMN_1361751	LOC293723	1.25	0.0204
ILMN_1363198	RGD1306682_predicted	1.25	0.0002
ILMN_1357229	Ccl2	1.25	0.0059
ILMN_1350480	S100a6	1.25	0.0085
ILMN_1364880	Aprt	1.25	0.0008
ILMN_1366030	Cacna2d1	0.75	0.0011
ILMN_1371786	Cpeb4_predicted	0.75	0.0004
ILMN_1366783	Vps13d_predicted	0.75	0.0005
ILMN_1362620	Pdp2	0.75	0.0013
ILMN_1356548	LOC501548	0.73	0.0056
ILMN_1361484	Nnt	0.72	0.0001
ILMN_1371120	LOC501637	0.66	0.0006
ILMN_1650171	Ttn	0.64	0.0001

**Table 4:** Summary of gene ontology (GO) categories of the main biological processes overrepresented among the differentially expressed genes in 24‐week‐old rats between sedentary versus early exercise and sedentary versus later exercise (*P* < 0.05)

GO term	Sedentary versus early exercise		Sedentary versus later exercise	
	Count	*P* value	Count	*P* value
Biological process	/34		/231	
GO:0006414˜translational elongation	3	0.0275	12	0.0000
GO:0070482˜response to oxygen levels	4	0.0176	13	0.0005
GO:0051240˜positive regulation of multicellular organismal process	6	0.0005	10	0.0487
GO:0016477˜cell migration	4	0.0330	11	0.0266
GO:0030595~leukocyte chemotaxis	3	0.0045	5	0.0051
GO:0060326~cell chemotaxis	3	0.0053	5	0.0068
GO:0050900~leukocyte migration	3	0.0105	5	0.0230
GO:0030155~regulation of cell adhesion	3	0.0422	7	0.0270
GO:0045785~positive regulation of cell adhesion	3	0.0120	5	0.0286
GO:0043066˜negative regulation of apoptosis	5	0.0123	0	N/A
GO:0043069~negative regulation of programmed cell death	5	0.0129	0	N/A
GO:0060548~negative regulation of cell death	5	0.0131	0	N/A
GO:0006935~chemotaxis	3	0.0214	0	N/A
GO:0042330~taxis	3	0.0214	0	N/A
GO:0048010~vascular endothelial growth factor receptor signaling pathway	2	0.0296	0	N/A
GO:0032760~positive regulation of tumor necrosis factor production	2	0.0349	0	N/A
GO:0009725~response to hormone stimulus	5	0.0487	0	N/A
GO:0032989~cellular component morphogenesis	0	N/A	18	0.0008
GO:0045792~negative regulation of cell size	0	N/A	8	0.0013
GO:0006412˜translation	0	N/A	19	0.0014
GO:0007568~aging	0	N/A	10	0.0023
GO:0007507˜heart development	0	N/A	12	0.0025
GO:0030030~cell projection organization	0	N/A	16	0.0038
GO:0032868~response to insulin stimulus	0	N/A	9	0.0039
GO:0030308~negative regulation of cell growth	0	N/A	7	0.0039
GO:0032535~regulation of cellular component size	0	N/A	12	0.0047
GO:0048738~cardiac muscle tissue development	0	N/A	6	0.0048
GO:0001558˜regulation of cell growth	0	N/A	10	0.0050
GO:0010243~response to organic nitrogen	0	N/A	8	0.0055
GO:0010959~regulation of metal ion transport	0	N/A	7	0.0056
GO:0000902~cell morphogenesis	0	N/A	15	0.0056
GO:0009719~response to endogenous stimulus	0	N/A	21	0.0065
GO:0048812~neuron projection morphogenesis	0	N/A	11	0.0081
GO:0030029~actin filament‐based process	0	N/A	10	0.0083
GO:0051924~regulation of calcium ion transport	0	N/A	6	0.0083
GO:0008361~regulation of cell size	0	N/A	10	0.0089
GO:0010769~regulation of cell morphogenesis involved in differentiation	0	N/A	7	0.0095
GO:0080135~regulation of cellular response to stress	0	N/A	7	0.0095
GO:0007242~intracellular signaling cascade	0	N/A	29	0.0096
GO:0051130~positive regulation of cellular component organization	0	N/A	10	0.0098
GO:0043269~regulation of ion transport	0	N/A	7	0.0104
GO:0022604~regulation of cell morphogenesis	0	N/A	8	0.0116
GO:0006955~immune response	0	N/A	16	0.0129
GO:0045926~negative regulation of growth	0	N/A	7	0.0131
GO:0010035~response to inorganic substance	0	N/A	12	0.0136
GO:0031175~neuron projection development	0	N/A	12	0.0155
GO:0048858~cell projection morphogenesis	0	N/A	11	0.0157
GO:0060537~muscle tissue development	0	N/A	8	0.0163
GO:0033043~regulation of organelle organization	0	N/A	9	0.0171
GO:0000904~cell morphogenesis involved in differentiation	0	N/A	11	0.0175
GO:0048667~cell morphogenesis involved in neuron differentiation	0	N/A	10	0.0175
GO:0030036~actin cytoskeleton organization	0	N/A	9	0.0187
GO:0006006~glucose metabolic process	0	N/A	9	0.0193
GO:0032990~cell part morphogenesis	0	N/A	11	0.0203
GO:0031344~regulation of cell projection organization	0	N/A	7	0.0208
GO:0019882~antigen processing and presentation	0	N/A	6	0.0209
GO:0042493~response to drug	0	N/A	13	0.0227
GO:0040008~regulation of growth	0	N/A	12	0.0274
GO:0006928~cell motion	0	N/A	15	0.0279
GO:0002252~immune effector process	0	N/A	7	0.0280
GO:0010033~response to organic substance	0	N/A	27	0.0298
GO:0000302~response to reactive oxygen species	0	N/A	6	0.0309
GO:0007155~cell adhesion	0	N/A	16	0.0314
GO:0022610~biological adhesion	0	N/A	16	0.0314
GO:0005996~monosaccharide metabolic process	0	N/A	10	0.0319
GO:0009611~response to wounding	0	N/A	15	0.0323
GO:0048666~neuron development	0	N/A	13	0.0335
GO:0045859~regulation of protein kinase activity	0	N/A	11	0.0348
GO:0014706~striated muscle tissue development	0	N/A	7	0.0390
GO:0019318~hexose metabolic process	0	N/A	9	0.0403
GO:0007517~muscle organ development	0	N/A	8	0.0427
GO:0030001~metal ion transport	0	N/A	14	0.0442
GO:0043549~regulation of kinase activity	0	N/A	11	0.0460

In contrast, at 24 weeks of age there were 280 differentially expressed genes following later exercise (compared to sedentary), with 196 having increased expression and 84 having reduced expression (data not shown; for a summary of the 36 genes with a fold change <0.60 or >1.40‐fold, see Table 5.) Gene ontology (GO) annotations revealed 10–20 genes clustered significantly to biological processes that included translational elongation, translation, response to oxygen levels, cell migration and morphogenesis, regulation of cell size, regulation of cell growth, and heart development (Table 4).

**Table 5:** Summary of DNA microarray differentially expressed genes in 24‐week‐old hearts from Control rats in the sedentary versus later exercise groups; <0.60 or >1.40 fold change. A fold change >1 indicates higher expression in later exercise rats. *N* = 7 rats per group

Illumina #	Gene symbol	Fold change Sedentary versus Later Ex	*P* value
ILMN_1359425	RGD1305975_predicted	1.91	0.0000
ILMN_1354070	isg12(b)	1.62	0.0028
ILMN_1368284	LOC501062	1.60	0.0000
ILMN_1361810	Reg3g	1.60	0.0059
ILMN_1363818	Ssr2_predicted	1.55	0.0001
ILMN_1376459	RT1‐Db1	1.48	0.0000
ILMN_1376479	Gstp1	1.47	0.0000
ILMN_1369599	Pf4	1.47	0.0000
ILMN_2038798	Actb	1.45	0.0067
ILMN_1355682	Cd63	1.45	0.0155
ILMN_1367740	Mt1a	1.44	0.0047
ILMN_1349244	Ptma	1.44	0.0003
ILMN_1349268	Rhoa	1.43	0.0012
ILMN_1360881	MGC112899	1.42	0.0036
ILMN_1371456	Cfd	1.42	0.0006
ILMN_1373973	RGD1565047_predicted	1.42	0.0001
ILMN_1350797	LOC300731	1.42	0.0001
ILMN_1370230	RGD1560513_predicted	1.41	0.0000
ILMN_1367581	RGD1560687_predicted	1.41	0.0040
ILMN_1376917	RT1‐M6‐2	1.41	0.0016
ILMN_1353276	RGD1559981_predicted	1.41	0.0008
ILMN_1350723	Rab3b	1.41	0.0004
ILMN_1364180	RGD1564883_predicted	1.40	0.0001
ILMN_1650955	Ifi27l	1.40	0.0001
ILMN_1352142	C1qb	1.40	0.0030
ILMN_1354975	RGD1563311_predicted	1.40	0.0000
ILMN_1366030	Cacna2d1	0.59	0.0000
ILMN_1374387	Itga1	0.58	0.0000
ILMN_1650171	Ttn	0.57	0.0000
ILMN_1362032	Ptprb_predicted	0.56	0.0000
ILMN_1350576	LOC290704	0.55	0.0000
ILMN_1352293	Bmpr2	0.53	0.0000
ILMN_1352642	Spp1	0.51	0.0110
ILMN_1364550	Sv2b	0.48	0.0003
ILMN_1356548	LOC501548	0.39	0.0000
ILMN_1371120	LOC501637	0.38	0.0000

### Real‐time PCR analysis

To validate our gene array data and include the Restricted and Reduced litter groups in our analysis, four differentially expressed genes identified from the array as having >1.25‐fold expression compared to sedentary in both early and later exercise groups were selected for real‐time PCR analysis in 24‐week‐old rats (Table 6).

**Table 6:** Summary of genes in the Control group from the DNA microarray in 24‐week‐old hearts from sedentary versus early exercise and sedentary versus later exercise rats that were selected for real‐time PCR analysis

Illumina #	Gene	FC sedentary versus early exercise	*P* value	FC sedentary versus laterexercise	*P* value
ILMN_1352642	Secreted phosphoprotein 1 (Spp1, also known as Osteopontin)	1.91	0.0126	0.51	0.0110
ILMN_1349268	Ras homolog gene family, member A (Rhoa)	1.50	0.0003	1.43	0.0012
ILMN_2038798	Actin, beta (Actb)	1.34	0.0255	1.45	0.0067
ILMN_1361581	Extracellular superoxide dismutase (Sod3)	1.26	0.0020	1.31	0.0005
ILMN_1372305	Cytochrome c oxidase subunit IV isoform 1 (Cox4i1)	1.16	0.0873	1.33	0.0026
ILMN_1350890	Creatine kinase, muscle (Ckm)	1.20	0.0192	1.30	0.0015

A fold change (FC) > 1 indicates higher expression in early or later exercise rats compared to sedentary.

**Figure 4:**
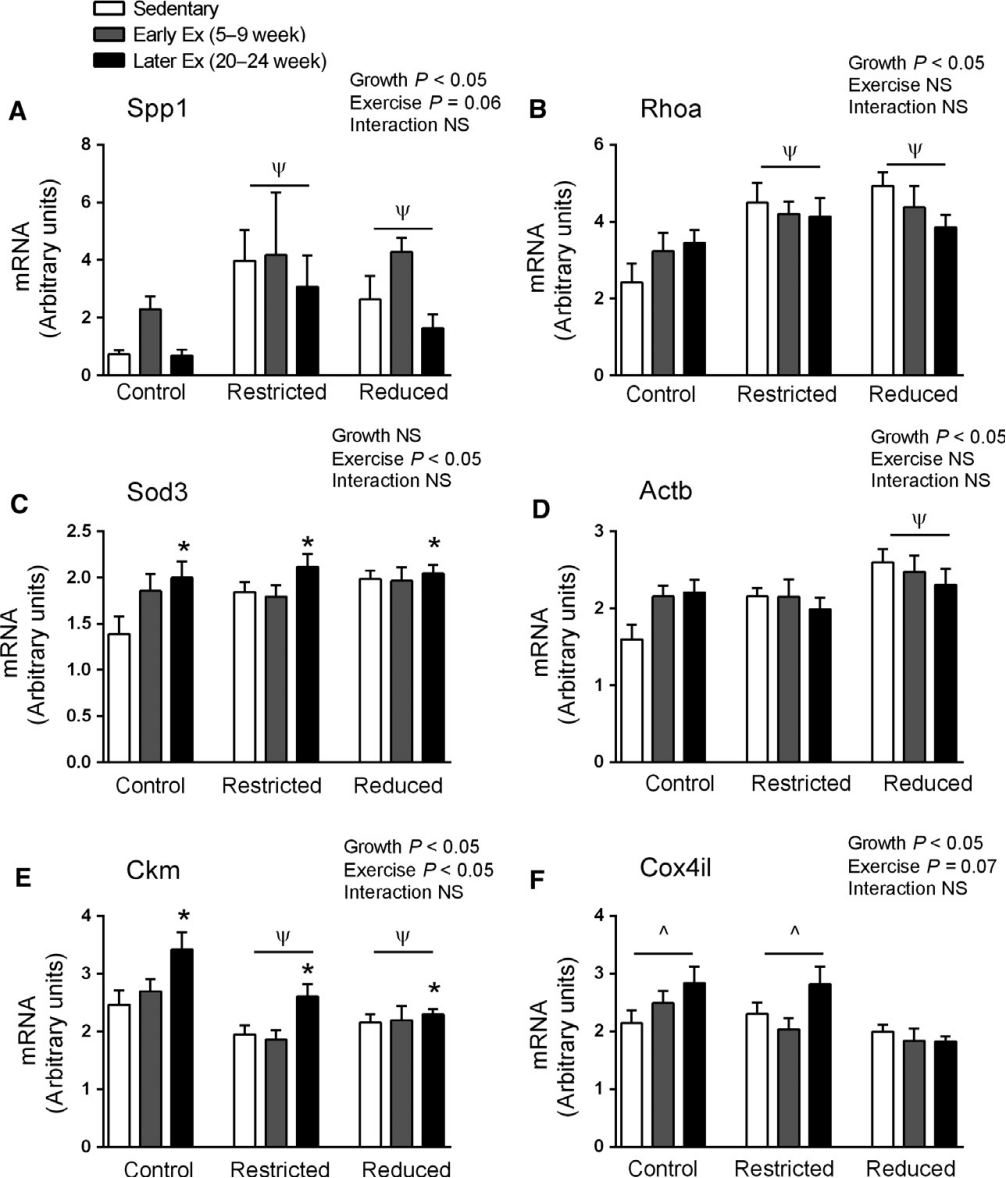
Whole heart mRNA levels at 24 weeks of age. Data are presented as mean ± SEM (*n* = 7–10/group). Early exercise (Early Ex) and later exercise (Later Ex). *P <* *0.05 versus Sedentary (main effect of exercise). ^Ψ^ *P *<* *0.05 versus Control, ^*P *<* *0.05 versus Reduced (Bonferroni comparisons of main effects).

Spp1 plays a role in the extracellular matrix binding and its upregulation has been linked to cardiac hypertrophy in response to chronic pressure overload (Xie et al. 2004). Spp1 is also proposed to play a key role in skeletal muscle injury repair by promoting myoblast proliferation (Pagel et al. 2014), although its role in cardiomyocyte repair and/or proliferation is unclear. Spp1 mRNA was significantly higher in both Restricted and Reduced litter rats (*P *<* *0.05, main effect for growth, Fig. 4A) and there was an overall tendency for endurance training (*P *=* *0.06) to increase Spp1 mRNA (Fig. 4A). Rhoa is a small G‐protein known to play a role in the development of cardiac hypertrophy and may have a cardioprotective role following ischemia reperfusion injury (Miyamoto et al. 2010). Compared to Control rats, Rhoa was significantly higher in both Restricted and Reduced litter rats (*P *<* *0.05, main effect for growth, Fig. 4B). Sod3 is a key antioxidant enzyme and cardioprotective against myocardial infarction (Konkalmatt et al. 2013). Sod3 mRNA was significantly higher following later exercise (*P *<* *0.05, main effect for exercise, Fig. 4C). Also, Rhoa, Actb, and Sod3 have all previously been shown to be upregulated following exercise‐induced cardiac hypertrophy (Iemitsu et al. 2005) (Strom et al. 2005; Galindo et al. 2009). Reduced litter rats had significantly higher Actb mRNA than the Control and Restricted rats (*P *<* *0.05, main effect for growth, Fig. 4D).

Ckm was significantly lower in Restricted and Reduced litter rats than Control rats (*P* < 0.05, main effect for growth, Fig. 4E). Later exercise significantly increased Ckm mRNA levels (*P *<* *0.05, main effect for exercise, Fig. 4E). Reduced litter rats had significantly lower Cox4il mRNA (*P *<* *0.05, main effect for growth, Fig. 4F), with exercise tending to increase Cox4il mRNA (*P *=* *0.07, main effect for exercise, Fig. 4F).

## Discussion

The major finding of this study is that when juvenile (5–9 weeks old) rats perform endurance training for just a few weeks and then remain inactive they display significantly elevated heart mass in adult life. The effect of the early exercise was as potent as exercise in later life when the heart mass measurements were made immediately after the exercise training period. Since it is unlikely that the acute effects of the early exercise were sustained for 4 months, these results suggest that there were epigenetics effects of early‐life exercise that benefit the adult heart. However, our mRNA analyses did not reveal any obvious candidates to explain these epigenetic effects. Interestingly, though, early exercise was associated with elevated phosphorylation of Akt Ser^473^ in adulthood, suggesting that the observation of elevated heart mass is physiological and not pathological hypertrophy (Bernardo et al. 2010).

Many studies in adult rats and adult humans have shown that endurance training increases heart mass, but this effect is only temporary and lost several weeks after training is stopped (Leon and Bloor 1976; Ehsani et al. 1978; Hickson et al. 1979, 1983; Frenzel et al. 1988; Kemi et al. 2004). Therefore, it would be expected that the increase in heart mass following endurance training is also temporary for young rats and humans. However, to our knowledge, no one has previously examined this. In the current study, 24‐week‐old rats that performed early exercise had significantly higher heart mass compared with the whole‐life sedentary rats. This increase in relative heart mass after early exercise was due to a larger absolute heart weight, since body weight and fat mass in adulthood were not altered by early exercise (Laker et al. 2012). Furthermore, this ~10% increase in heart mass (cardiac hypertrophy) is physiologically meaningful, as heart mass typically increases ~10–30% following endurance training in rats (Hickson et al. 1979, 1983; Lennon et al. 2004). In addition, total protein and RNA concentrations were unchanged (Table 1) which is consistent with previous endurance training studies (Hickson et al. 1979; Beyer et al. 1984). It is not known whether early exercise impacts positively on important cardiac parameters that are indicative of physiological cardiac hypertrophy, such as cardiac dimensions (i.e., increased left ventricle mass and thickness), cardiac function (i.e., increased stroke volume and whole‐body maximal oxygen uptake), and cardiomyocyte size and number (Leon and Bloor 1976; Ehsani et al. 1978; Hickson et al. 1979, 1983; Frenzel et al. 1988; Kemi et al. 2004). Further studies are now required to investigate these parameters.

The IGF1‐PI3K(p110*α*)‐Akt signaling pathway has a critical role in regulating physiological (but not pathological) cardiac growth (reviewed in (Bernardo et al. 2010)). In the present study the later exercise group acts as an exercise “control” group, since heart mass is well known in adult rats to be elevated following several weeks of endurance training (Leon and Bloor 1976; Ehsani et al. 1978; Hickson et al. 1979, 1983; Frenzel et al. 1988; Kemi et al. 2004). Therefore, it was surprising that phosphorylation of Akt Ser^473^ was not increased in the later exercise group despite the increased heart mass. However, to avoid the residual effects of the last bout of exercise training, rats in later exercise group were killed 72 h post exercise. Therefore, it is possible that Akt Ser^473^ phosphorylation had returned to its basal levels by this stage. Indeed, studies reporting elevated phosphorylation of Akt Ser^473^ typically examine hearts within 24–48 h of the last bout of exercise (Kemi et al. 2008; Dobrzyn et al. 2013; Ma et al. 2013). Furthermore, cardiac phosphorylation of Akt Ser^473^ has also been reported to increase in the initial weeks of endurance training and then decline to basal levels (Konhilas et al. 2004). Thus, given the lack of Akt upregulation in the later exercise group, the increased phosphorylation and protein abundance of Akt in the early exercise groups was a surprising finding and suggests potentially long‐term upregulation and programming of the protein synthesis pathway. However, these results should be viewed with caution, since they were only significantly higher compared to the later exercise group, not compared with the sedentary group. Nevertheless, future studies are now warranted to examine if early exercise can lead to long‐term upregulation of cardiac protein synthesis pathways.

Microarray analysis was conducted to investigate the molecular pathways that may be responsible for endurance training leading to increases in heart mass. Since the increased heart mass response to training was similar in the Control, Reduced litter, and Restricted groups, we performed microarray in the Control (sedentary, early, and later exercise) animals only. Consistent with previous microarray studies, several weeks of endurance training in the later exercise (compared to sedentary), adult rats upregulated large numbers of genes related to physiological cardiac growth and energy metabolism (Diffee et al. 2003; Strom et al. 2005). However, with regard to the effects of early exercise (vs. sedentary) in adult rats, there were only a small number of genes significantly clustered to biological processes that might potentially be regulating heart size such as translational elongation, cell migration, and apoptosis. Further validation of some of these differentially expressed genes via real‐time PCR to also include Control, Restricted, and Reduced litter rats did not reveal any long‐term effects of early exercise on the expression of any of these genes individually. Therefore, despite potential long‐term reprogramming of early exercise on heart mass, its regulation does not appear to be at the level of gene expression.

The delayed catch up growth in Reduced litter offspring in the current study (Laker et al. 2012) was not accompanied by elevations in systolic blood pressure in adulthood, which is contrary to previous findings in other cohorts of WKY rats from our laboratory (Wlodek et al. 2007, 2008; Black et al. 2012). Similarly, others have shown that maternal protein restriction in WKY rats does not always result in higher blood pressure, even in the presence of a kidney nephron deficit (Zimanyi et al. 2004). It is possible that the differences between the degree and timing of growth patterns in both Restricted and Reduced litter groups compared with previous studies may account for these observations. Furthermore, the normal blood pressure observed in the current study in Restricted and Reduced litter offspring may be a consequence of lower exposure to stress due to the considerably higher levels of animal handling than our previous studies. Indeed, animals in the current study were subject to daily exercise and weighing during exercise periods, food intake measurements, monthly blood pressure, weight, and body dimension recordings. Furthermore, it has been reported that regular handling of rats from a young age reduces the adult adrenocoritcal stress response (Meaney et al. 1991). Therefore, the almost daily handling of rats in the current study may have reduced the stress response to subsequent handling events in later life and prevented or delayed the onset of elevated blood pressure that was programmed in early life in Restricted and Reduced litter offspring.

In conclusion, in all groups, exercise early in life increased adult heart mass despite the animals being sedentary for ~4 months. Greater cardiac phosphorylation of Akt Ser^473^ in adulthood supports an upregulation of processes involved in cardiac growth due to exercise in early life. However, further work is required to establish the molecular mechanisms responsible for these novel findings, since they do not appear to be regulated at the level of gene expression. The findings of the present study suggest that the juvenile period of life is a stage of developmental plasticity that is amenable to long‐term, beneficial cardiac programming by short‐term endurance training.

## Conflict of Interest

None declared.

## References

[phy212720-bib-0001] Barker, D. J. , C. Osmond , J. Golding , D. Kuh , and M. E. Wadsworth . 1989 Growth in utero, blood pressure in childhood and adult life, and mortality from cardiovascular disease. BMJ 298:564–567. 2495113 10.1136/bmj.298.6673.564 PMC1835925

[phy212720-bib-0002] Bernardo, B. C. , K. L. Weeks , L. Pretorius , and J. R. McMullen . 2010 Molecular distinction between physiological and pathological cardiac hypertrophy: experimental findings and therapeutic strategies. Pharmacol. Ther. 128:191–227. 20438756 10.1016/j.pharmthera.2010.04.005

[phy212720-bib-0003] Beyer, R. E. , P. G. Morales‐Corral , B. J. Ramp , K. R. Kreitman , M. J. Falzon , S. Y. Rhee , et al. 1984 Elevation of tissue coenzyme Q (ubiquinone) and cytochrome c concentrations by endurance exercise in the rat. Arch. Biochem. Biophys. 234:323–329. 6093695 10.1016/0003-9861(84)90277-7

[phy212720-bib-0004] Black, M. J. , A. L. Siebel , O. Gezmish , K. Moritz , and M. E. Wlodek . 2012 Normal lactational environment restores cardiomyocyte number after uteroplacental insufficiency: implications for the preterm neonate. Am. J. Physiol. 302:R1101–R1110. 10.1152/ajpregu.00030.2012 PMC3362141 22403799

[phy212720-bib-0005] Diffee, G. M. , E. A. Seversen , T. D. Stein , and J. A. Johnson . 2003 Microarray expression analysis of effects of exercise training: increase in atrial MLC‐1 in rat ventricles. Am. J. Physiol. Heart Circ. Physiol. 284:H830–H837. 12424097 10.1152/ajpheart.00761.2002

[phy212720-bib-0006] Dobrzyn, P. , A. Pyrkowska , M. K. Duda , T. Bednarski , M. Maczewski , J. Langfort , et al. 2013 Expression of lipogenic genes is upregulated in the heart with exercise training‐induced but not pressure overload‐induced left ventricular hypertrophy. Am. J. Physiol. Endocrinol Metab. 304:E1348–E1358. 23632628 10.1152/ajpendo.00603.2012

[phy212720-bib-0007] Du, P. , W. A. Kibbe , and S. M. Lin . 2008 lumi: a pipeline for processing Illumina microarray. Bioinformatics 24:1547–1548. 18467348 10.1093/bioinformatics/btn224

[phy212720-bib-0008] Echegaray, M. , and M. A. Rivera . 2001 Role of creatine kinase isoenzymes on muscular and cardiorespiratory endurance: genetic and molecular evidence. Sports Med. 31:919–934. 11708401 10.2165/00007256-200131130-00003

[phy212720-bib-0009] Ehsani, A. A. , J. M. Hagberg , and R. C. Hickson . 1978 Rapid changes in left ventricular dimensions and mass in response to physical conditioning and deconditioning. Am. J. Cardiol. 42:52–56. 677037 10.1016/0002-9149(78)90984-0

[phy212720-bib-0010] Eriksson, J. , T. Forsen , J. Tuomilehto , C. Osmond , and D. Barker . 2000 Fetal and childhood growth and hypertension in adult life. Hypertension 36:790–794. 11082144 10.1161/01.hyp.36.5.790

[phy212720-bib-0011] Fagard, R. H. 2005 Effects of exercise, diet and their combination on blood pressure. J. Hum. Hypertens. 19(Suppl. 3):S20–S24. 16302006 10.1038/sj.jhh.1001956

[phy212720-bib-0012] Fagard, R. H. 2006 Exercise is good for your blood pressure: effects of endurance training and resistance training. Clin. Exp. Pharmacol. Physiol. 33:853–856. 16922820 10.1111/j.1440-1681.2006.04453.x

[phy212720-bib-0013] Fagard, R. H. , and V. A. Cornelissen . 2007 Effect of exercise on blood pressure control in hypertensive patients. Eur. J. Cardiovasc. Prev. Rehabil. 14:12–17. 17301622 10.1097/HJR.0b013e3280128bbb

[phy212720-bib-0014] Fernandes, R. A. , and A. Zanesco . 2010 Early physical activity promotes lower prevalence of chronic diseases in adulthood. Hypertens. Res. 33:926–931. 20574424 10.1038/hr.2010.106

[phy212720-bib-0015] Frenzel, H. , B. Schwartzkopff , W. Holtermann , H. G. Schnurch , A. Novi , and W. Hort . 1988 Regression of cardiac hypertrophy: morphometric and biochemical studies in rat heart after swimming training. J. Mol. Cell. Cardiol. 20:737–751. 2975711 10.1016/s0022-2828(88)80018-x

[phy212720-bib-0016] Galindo, C. L. , M. A. Skinner , M. Errami , L. D. Olson , D. A. Watson , J. Li , et al. 2009 Transcriptional profile of isoproterenol‐induced cardiomyopathy and comparison to exercise‐induced cardiac hypertrophy and human cardiac failure. BMC Physiol. 9:23. 20003209 10.1186/1472-6793-9-23 PMC2799380

[phy212720-bib-0017] Green, D. J. , A. Spence , J. R. Halliwill , N. T. Cable , and D. H. Thijssen . 2011 Exercise and vascular adaptation in asymptomatic humans. Exp. Physiol. 96:57–70. 20971800 10.1113/expphysiol.2009.048694

[phy212720-bib-0018] Hickson, R. C. , G. T. Hammons , and J. O. Holloszy . 1979 Development and regression of exercise‐induced cardiac hypertrophy in rats. Am. J. Physiol. 236:H268–H272. 217279 10.1152/ajpheart.1979.236.2.H268

[phy212720-bib-0019] Hickson, R. C. , T. M. Galassi , and K. A. Dougherty . 1983 Repeated development and regression of exercise‐induced cardiac hypertrophy in rats. J. Appl. Physiol. (1985) 54:794–797. 10.1152/jappl.1983.54.3.794 6221005

[phy212720-bib-0020] Huang, D. W. , B. T. Sherman , and R. A. Lempicki . 2008 Systematic and integrative analysis of large gene lists using DAVID bioinformatics resources. Nat. Protoc. 4:44–57. 10.1038/nprot.2008.211 19131956

[phy212720-bib-0021] da Huang, W. , B. T. Sherman , and R. A. Lempicki . 2009 Bioinformatics enrichment tools: paths toward the comprehensive functional analysis of large gene lists. Nucleic Acids Res. 37:1–13. 19033363 10.1093/nar/gkn923 PMC2615629

[phy212720-bib-0022] Iemitsu, M. , S. Maeda , T. Miyauchi , M. Matsuda , and H. Tanaka . 2005 Gene expression profiling of exercise‐induced cardiac hypertrophy in rats. Acta Physiol. Scand. 185:259–270. 16266368 10.1111/j.1365-201X.2005.01494.x

[phy212720-bib-0023] Kemi, O. J. , P. M. Haram , U. Wisloff , and O. Ellingsen . 2004 Aerobic fitness is associated with cardiomyocyte contractile capacity and endothelial function in exercise training and detraining. Circulation 109:2897–2904. 15173028 10.1161/01.CIR.0000129308.04757.72

[phy212720-bib-0024] Kemi, O. J. , M. Ceci , U. Wisloff , S. Grimaldi , P. Gallo , G. L. Smith , et al. 2008 Activation or inactivation of cardiac Akt/mTOR signaling diverges physiological from pathological hypertrophy. J. Cell. Physiol. 214:316–321. 17941081 10.1002/jcp.21197

[phy212720-bib-0025] Konhilas, J. P. , A. H. Maass , S. W. Luckey , B. L. Stauffer , E. N. Olson , and L. A. Leinwand . 2004 Sex modifies exercise and cardiac adaptation in mice. Am. J. Physiol. Heart. Circ. Physiol. 287:H2768–H2776. 15319208 10.1152/ajpheart.00292.2004 PMC2637113

[phy212720-bib-0026] Konkalmatt, P. R. , R. J. Beyers , D. M. O'Connor , Y. Xu , M. E. Seaman , and B. A. French . 2013 Cardiac‐selective expression of extracellular superoxide dismutase after systemic injection of Adeno‐associated virus 9 protects the heart against post‐myocardial infarction left ventricular remodeling. Circ. Cardiovasc. Imaging 6:478–486. 23536266 10.1161/CIRCIMAGING.112.000320 PMC3762576

[phy212720-bib-0027] Laker, R. C. , L. A. Gallo , M. E. Wlodek , A. L. Siebel , G. D. Wadley , and G. K. McConell . 2011 Short‐term exercise training early in life restores deficits in pancreatic beta‐cell mass associated with growth restriction in adult male rats. Am. J. Physiol. 301:E931–E940. 10.1152/ajpendo.00114.2011 21810930

[phy212720-bib-0028] Laker, R. C. , M. E. Wlodek , G. D. Wadley , L. A. Gallo , P. J. Meikle , and G. K. McConell . 2012 Exercise early in life in rats born small does not normalize reductions in skeletal muscle PGC‐1*α* in adulthood. Am. J. Physiol. 302:E1221–E1230. 10.1152/ajpendo.00583.2011 22354784

[phy212720-bib-0029] Lennon, S. L. , J. Quindry , K. L. Hamilton , J. French , J. Staib , J. L. Mehta , et al. 2004 Loss of exercise‐induced cardioprotection after cessation of exercise. J. Appl. Physiol. (1985) 96:1299–1305. 14672968 10.1152/japplphysiol.00920.2003

[phy212720-bib-0030] Leon, A. S. , and C. M. Bloor . 1976 The effect of complete and partial deconditioning on exercise‐induced cardiovascular changes in the rat. Adv. Cardiol. 18:81–92. 136175 10.1159/000399514

[phy212720-bib-0031] Lin, S. M. , P. Du , W. Huber , and W. A. Kibbe . 2008 Model‐based variance‐stabilizing transformation for Illumina microarray data. Nucleic Acids Res. 36:e11. 18178591 10.1093/nar/gkm1075 PMC2241869

[phy212720-bib-0032] Lundby, C. , N. Nordsborg , K. Kusuhara , K. M. Kristensen , P. D. Neufer , and H. Pilegaard . 2005 Gene expression in human skeletal muscle: alternative normalization method and effect of repeated biopsies. Eur. J. Appl. Physiol. 95:351–360. 16151837 10.1007/s00421-005-0022-7

[phy212720-bib-0033] Ma, Z. , J. Qi , S. Meng , B. Wen , and J. Zhang . 2013 Swimming exercise training‐induced left ventricular hypertrophy involves microRNAs and synergistic regulation of the PI3K/AKT/mTOR signaling pathway. Eur. J. Appl. Physiol. 113:2473–2486. 23812090 10.1007/s00421-013-2685-9

[phy212720-bib-0034] McMullen, J. R. , and G. L. Jennings . 2007 Differences between pathological and physiological cardiac hypertrophy: novel therapeutic strategies to treat heart failure. Clin. Exp. Pharmacol. Physiol. 34:255–262. 17324134 10.1111/j.1440-1681.2007.04585.x

[phy212720-bib-0035] Meaney, M. J. , J. B. Mitchell , D. H. Aitken , S. Bhatnagar , S. R. Bodnoff , L. J. Iny , et al. 1991 The effects of neonatal handling on the development of the adrenocortical response to stress: implications for neuropathology and cognitive deficits in later life. Psychoneuroendocrinology 16:85–103. 1961847 10.1016/0306-4530(91)90072-2

[phy212720-bib-0036] Miyamoto, S. , D. Del Re , S. Xiang , X. Zhao , G. Florholmen , and J. Brown . 2010 Revisited and revised: is RhoA always a villain in cardiac pathophysiology? J. Cardiovasc. Transl. Res. 3:330–343. 20559774 10.1007/s12265-010-9192-8 PMC3005405

[phy212720-bib-0037] Mollica, J. P. , J. S. Oakhill , G. D. Lamb , and R. M. Murphy . 2009 Are genuine changes in protein expression being overlooked? Reassessing Western blotting Anal. Biochem. 386:270–275. 19161968 10.1016/j.ab.2008.12.029

[phy212720-bib-0038] O'Dowd, R. , J. C. Kent , J. M. Moseley , and M. E. Wlodek . 2008 Effects of uteroplacental insufficiency and reducing litter size on maternal mammary function and postnatal offspring growth. Am. J. Physiol. 294:R539–R548. 10.1152/ajpregu.00628.2007 18077510

[phy212720-bib-0039] Pagel, C. N. , D. K. Wasgewatte Wijesinghe , N. Taghavi Esfandouni , and E. J. Mackie . 2014 Osteopontin, inflammation and myogenesis: influencing regeneration, fibrosis and size of skeletal muscle. J. Cell Commun. Signal. 8:95–103. 24318932 10.1007/s12079-013-0217-3 PMC4063988

[phy212720-bib-0040] Pluim, B. M. , A. H. Zwinderman , A. van der Laarse , and E. E. van der Wall . 2000 The Athlete's Heart: a meta‐analysis of cardiac structure and function. Circulation 101:336–344. 10645932 10.1161/01.cir.101.3.336

[phy212720-bib-0041] Qi, Z. , J. He , Y. Su , Q. He , J. Liu , L. Yu , et al. 2011 Physical exercise regulates p53 activity targeting SCO2 and increases mitochondrial COX biogenesis in cardiac muscle with age. PLoS ONE 6:e21140. 21750704 10.1371/journal.pone.0021140 PMC3131270

[phy212720-bib-0042] Siebel, A. L. , A. Mibus , M. J. De Blasio , K. T. Westcott , M. J. Morris , L. Prior , et al. 2008 Improved lactational nutrition and postnatal growth ameliorates impairment of glucose tolerance by uteroplacental insufficiency in male rat offspring. Endocrinology 149:3067–3076. 18339706 10.1210/en.2008-0128

[phy212720-bib-0043] Storgaard, H. , P. Poulsen , C. Ling , L. Groop , and A. A. Vaag . 2006 Genetic and nongenetic determinants of skeletal muscle glucose transporter 4 messenger ribonucleic acid levels and insulin action in twins. J. Clin. Endocrinol. Metab. 91:702–708. 16291707 10.1210/jc.2005-1172

[phy212720-bib-0044] Strom, C. C. , M. Aplin , T. Ploug , T. E. Christoffersen , J. Langfort , M. Viese , et al. 2005 Expression profiling reveals differences in metabolic gene expression between exercise‐induced cardiac effects and maladaptive cardiac hypertrophy. FEBS J. 272:2684–2695. 15943803 10.1111/j.1742-4658.2005.04684.x

[phy212720-bib-0045] Tipton, C. M. , J. M. Overton , E. B. Pepin , J. G. Edwards , J. Wegner , and E. M. Youmans . 1987 Influence of exercise training on resting blood pressures of Dahl rats. J. Appl. Physiol. (1985) 63:342–346. 3624135 10.1152/jappl.1987.63.1.342

[phy212720-bib-0046] Tipton, C. M. , L. A. Sebastian , J. M. Overton , C. R. Woodman , and S. B. Williams . 1991 Chronic exercise and its hemodynamic influences on resting blood pressure of hypertensive rats. J. Appl. Physiol. (1985) 71:2206–2210. 1778914 10.1152/jappl.1991.71.6.2206

[phy212720-bib-0047] Tsirka, A. E. , E. M. Gruetzmacher , D. E. Kelley , V. H. Ritov , S. U. Devaskar , and R. H. Lane . 2001 Myocardial gene expression of glucose transporter 1 and glucose transporter 4 in response to uteroplacental insufficiency in the rat. J. Endocrinol. 169:373–380. 11312153 10.1677/joe.0.1690373

[phy212720-bib-0048] Vickers, M. H. , B. H. Breier , W. S. Cutfield , P. L. Hofman , and P. D. Gluckman . 2000 Fetal origins of hyperphagia, obesity, and hypertension and postnatal amplification by hypercaloric nutrition. Am. J. Physiol. 279:E83–E87. 10.1152/ajpendo.2000.279.1.E83 10893326

[phy212720-bib-0049] Wadley, G. D. , and G. K. McConell . 2010 High‐dose antioxidant vitamin C supplementation does not prevent acute exercise‐induced increases in markers of skeletal muscle mitochondrial biogenesis in rats. J. Appl. Physiol. (1985) 108:1719–1726. 20395544 10.1152/japplphysiol.00127.2010

[phy212720-bib-0050] Wadley, G. D. , G. K. McConell , C. A. Goodman , A. L. Siebel , K. T. Westcott , and M. E. Wlodek . 2013 Growth restriction in the rat alters expression of metabolic genes during postnatal cardiac development in a sex‐specific manner. Physiol. Genomics 45:99–105. 23232075 10.1152/physiolgenomics.00095.2012

[phy212720-bib-0051] Wlodek, M. E. , A. Mibus , A. Tan , A. L. Siebel , J. A. Owens , and K. M. Moritz . 2007 Normal lactational environment restores nephron endowment and prevents hypertension after placental restriction in the rat. J. Am. Soc. Nephrol. 18:1688–1696. 17442788 10.1681/ASN.2007010015

[phy212720-bib-0052] Wlodek, M. E. , K. Westcott , A. L. Siebel , J. A. Owens , and K. M. Moritz . 2008 Growth restriction before or after birth reduces nephron number and increases blood pressure in male rats. Kidney Int. 74:187–195. 18432184 10.1038/ki.2008.153

[phy212720-bib-0053] Xie, Z. , M. Singh , and K. Singh . 2004 Osteopontin modulates myocardial hypertrophy in response to chronic pressure overload in mice. Hypertension 44:826–831. 15534078 10.1161/01.HYP.0000148458.03202.48

[phy212720-bib-0054] Zimanyi, M. A. , J. F. Bertram , and M. J. Black . 2004 Does a nephron deficit in rats predispose to salt‐sensitive hypertension? Kidney Blood Press. Res. 27:239–247. 15273426 10.1159/000079868

